# High diagnostic yield of electromagnetic navigation bronchoscopy performed under cone beam CT guidance: results of a randomized Belgian monocentric study

**DOI:** 10.1186/s12890-023-02492-7

**Published:** 2023-05-27

**Authors:** Benjamin Bondue, Olivier Taton, Fadi Tannouri, Nikita Van de Velde, Myriam Remmelink, Dimitri Leduc

**Affiliations:** 1 Department of Pneumology, Hôpital Erasme, Université Libre de Bruxelles, 808 Route de Lennik, 1070 Brussels, Belgium; 2 Department of Radiology, Hôpital Erasme, Université Libre de Bruxelles, Brussels, Belgium; 3 Department of Pathology, Hôpital Erasme, Université Libre de Bruxelles, Brussels, Belgium

**Keywords:** Electromagnetic navigation bronchoscopy, Cone beam computed tomography, Trans-bronchial lung cryobiopsy, Lung cancer, Pulmonary nodule, Bronchoscopy

## Abstract

**Background:**

With the increasing use of low dose CT scans, numerous pulmonary nodules are detected. As majority of them are benign, development of efficient non-surgical diagnostic intervention is mandatory. Electromagnetic navigation bronchoscopy (ENB) has been developed to reach difficult to access lesions. The aim of the present study was to compare the diagnostic yield of ENB procedures performed in a classical endoscopy suite or in a hybrid room equipped by a cone beam CT (CBCT).

**Methods:**

A monocentric randomized study was performed in the Erasme Hospital between January 2020 and December 2021. Lung nodules of maximum 30 mm of diameter were eligible. In both arms (endoscopy or CBCT suites), ENB, fluoroscopic guidance and a radial endobronchial ultrasound were used to reach the lesion. Then six trans-bronchial biopsies (TBB) and one trans-bronchial lung cryobiopsy (TBLC) were performed. Primary outcomes were the diagnostic yield and diagnostic accuracy of the procedure.

**Results:**

Forty-nine patients were randomized (24 in the endoscopy and 25 in the CBCT arms). The lesion size was 15,9 ± 4,6 mm and 16,6 ± 6,0 mm respectively (mean ± SD, *p* = NS). The diagnostic yield of ENB performed under CBCT guidance was 80% compared to 42% when performed in the endoscopy suite under standard fluoroscopic guidance (*p* < 0,05). Similarly, the diagnostic accuracy in the CBCT group was 87% compared to 54% for the endoscopy group (*p* < 0,05). Duration of the procedure in the CBCT and endoscopy arms was 80 ± 23 and 61 ± 13 min respectively (mean ± SD, *p* < 0,01). Performing TBLC in addition to TBB increased the diagnostic yield by 14% (17 and 12,5% in CBCT and endoscopy suites respectively, *p* = NS).

**Conclusion:**

This study highlighted the additional value to perform ENB procedure under CBCT guidance for small size (less than 2 cm of diameter) pulmonary nodules.

**Trial registration:**

Clinical trial registration number: NCT05257382.

## Background

Lung cancer is the cause of 1.80 million death in 2020 in the world and is the leading cause of cancer related death [1]. Recently two major randomized trials demonstrated the benefit of lung cancer screening strategies using low dose CT (LDCT) scans [2, 3]. With the increasing use of LDCT, numerous pulmonary nodules are detected. As majority of them are benign, non-invasive diagnostic procedures are mandatory to avoid unnecessary surgical resections [2].

Electromagnetic Navigation Bronchoscopy (ENB), a kind of GPS navigation system in the bronchi, aims to guide a catheter and biopsy instruments to the target. This technique improves the localization of the nodule and increases the diagnostic yield of biopsies with diagnostic yields ranging from 52 to 72,9% [4–7].

In our current practice, this technique is routinely used in an endoscopic suite in combination with X-ray fluoroscopic guidance, EBUS radial mini-probe (rEBUS) and trans-bronchial lung cryobiopsies (TBLCs) in addition to trans-bronchial biopsies (TBBs). The combination of TBLC to the ENB technique increased significantly the diagnostic yield of the ENB for the diagnosis of lung nodules of less than 20 mm [8].

Nevertheless, even if the combined use of these techniques increases the chance to diagnose lesions that are difficult to access, some of them are still not reached. As ENB procedure are based on pre-procedural HRCT performed a few days before the procedure (on awake patient holding breath at maximum inspiration), the exact position of the nodule could be slightly different between this pre-procedural HRCT and its real position during the procedure itself performed under general anesthesia (CT-to-body divergence). It could result into a mispositioning or a misorientation of biopsy instruments. Also, the lack of visible bronchus leading to the nodule (lack of bronchus sign) or the presence of tight angulations of the pathway reduce its accessibility. To solve these issues, ENB procedure can be performed in a hybrid room equipped by a cone beam computed tomography (CBCT), which is a variation of traditional computed tomography systems. The CBCT rotates around the patient, capturing data using a cone-shaped X-ray beam. These data are used to reconstruct a three-dimensional (3D) image, to obtain a real-time evaluation of the position of the ENB catheter and to visualize the lesion during X-ray fluoroscopy (enhanced fluoroscopy). Another advantage of CBCT is the use of trans-parenchymal access tools such as the “CrossCountry” catheter developed by Medtronic to reach the lesion if ENB alone does not allow a confident positioning of the catheter within the targeted lesion [9, 10].

To our knowledge, no study evaluated prospectively the benefit of performing ENB under CBCT guidance or under standard X-ray fluoroscopic guidance alone. Therefore, the aim of this study was to compare the diagnostic yield and diagnostic accuracy for malignancy of ENB procedures coupled to TBBs and TBLCs for the diagnosis of pulmonary nodule when performed either in a "standard" bronchoscopy suite or in a hybrid room equipped by a CBCT.

## Materials and methods

### Study design

A monocentric prospective and randomized trial was conducted between February 2020 and December 2021 in a tertiary academic hospital (Erasme hospital, Brussels, Belgium). The protocol was approved by the ethical committee of the institution (Ref. Nr: P2019/617), performed in accordance with the declaration of Helsinki, and registered on ClinicalTrials.gov (number: NCT05257382, date: 25/02/2022). Written informed consent was obtained from each participant.

Participants were randomly assigned in one of the following groups:

- ◦ "Endoscopy suite": ENB procedures were performed in an endoscopy room under standard X-ray fluoroscopy guidance.
- ◦ "CBCT suite": ENB procedures were performed in a room equipped with a CBCT (Philips Allura Clarity FD20 scanner (Philips, Best, The Netherlands)).

The allocation of the participants to each of these arms was performed by the principal investigator using a simple randomization technique by random draw (randomization 1:1).

### Inclusion and exclusion criteria

Adult patients (older than 18 years) were included if a lung biopsy was required for the diagnosis of a lung nodule of maximum 30 mm of diameter. Nodules could be either solid, part-solid or non-solid (ground glass opacity) without evidence of loco-regional or distant metastasis that could be biopsied, and no evidence for an infectious underlying disease. A multidisciplinary discussion was performed for each case before inclusion in the study in order to determine whether any other endoscopic or trans-thoracic procedure could be performed first, before considering ENB, or if patients should be referred directly to surgery.

Indeed, patient eligible for a direct surgical resection of the nodule (growing lesion on serial imaging, nodules that measures > 8 mm in diameter with a high clinical probability of malignancy or PET positivity) were preferentially allocated to surgery [11]. At the opposite, patients with an intermediate risk of malignancy and non-operable patients were preferentially allocated to the ENB procedure or trans-thoracic biopsy. Other exclusion criteria were a higher risk of bleeding (platelets count lower than 80,000/mm^3^, a systolic pulmonary arterial pressure (sPAP) higher than 45 mmHg at transthoracic cardiac ultrasonography (systematically performed before the procedure), prothrombin time international normalized ratio > 1.5, activated partial thromboplastin time > 35, uninterrupted anti-coagulant/anti-aggregant therapy), and the presence of any contraindication to general anesthesia as determined by the principal investigator (significant cardiac comorbidities, hypercapnia, severe hypoxia…).

### Endoscopic procedure

All procedures were performed under general anesthesia with the use of an endotracheal tube allowing the introduction of the Fogarty balloon in a separate channel (Bronchoflex-set, Rüsch.Inc, Duluth, MN, USA). To minimize the risk of atelectasis during the procedure, a positive end-expiratory pressure (PEEP) of minimum 5 cmH_2_0 (10 cmH_2_0 if tolerated) was maintained during the intervention as well as an FiO2 at its lowest tolerable level. Before the ENB procedure, an inspection bronchoscopy was performed using a flexible videobronchoscope (BF-1TH190, Olympus, Tokyo, Japan) and a Fogarty balloon was inserted into the segmental bronchus and inflated prophylactically after each TBLC for controlling possible bleeding. The ENB-based workflow utilized Medtronic SuperDimension platform (version 7.1; Medtronic, Minneapolis, MN, USA) following the manufacturer’s instruction. Briefly, a preprocedural segmented CT-based pathway was defined from the ENB planning platform. Navigation used catheters with a 180° preformed curvature (extended working channel/EdgeTM firm tip, Endobronchial Procedure Kit, 180 Catheter, Medtronic, Minneapolis, MN, USA). At the end of the ENB procedure, the position of the catheter was checked by fluoroscopy. For procedures performed in the hybrid room, CBCT scans and the Lung Suite software (Philips, Best, The Netherlands) were used to perform a segmentation of the lesion, to analyze the position and axis of the ENB catheter relative to the lesion, to perform enhanced fluoroscopy and the CrossCountry technique as required (see description below). If needed, the position of the catheter was modified accordingly and CBCT acquisitions repeated to improve the position of the navigation catheter in front of the lesion. During CBCT acquisition, a breath-hold with a PEEP of 10 cmH_2_0 using an adjustable pressure-limiting valve was performed to limit the risk of atelectasis and movement during the imaging. Additional breath-holds were also performed when the navigation catheter and biopsy tools are positioned under enhanced fluoroscopy. If the lesion remained unreachable, transparenchymal access was pursued using the CrossCountry technique according to the manufacture’s instruction (Medtronic, Minneapolis, MN, USA). Briefly, a small sharp tipped wire is deployed through the airway wall and into the parenchyma. Subsequently a cone-shaped dilator is advanced over the wire to the target. Then by the Seldinger technique, the extended working channel/Edge™ catheter is advanced over the dilator until the lesion is reached. The wire and dilator are then removed leaving access for biopsy tools though the extended working channel/Edge™ catheter.

In both groups, when the catheter seemed in correct position, the ENB sensor was exchanged for a rEBUS mini-probe imaging (UM- S20-17S Radial EBUS miniprobe; Olympus, Tokyo, Japan), and six forceps TBB were subsequently performed (Radial Jaw 4, Boston Scientific®, MA, USA). Then, a TBLC sample was obtained through a flexible cryoprobe of 115 cm in length and 1.9 mm (multiple-use) or 1,7 mm (single-use) in diameter (ERBE, Medizintechnik GmbH, Tubingen, Germany) inserted into the ENB catheter. Once in position, the probe was cooled for 8 s; then the catheter, the cryoprobe, and the bronchoscope were removed out of the airway, and the pre-positioned Fogarty balloon was inflated to control potential severe bleeding. For some patients in the CBCT group, no cryobiopsy was performed if the nodule appeared closer than expected to the pleura during CBCT acquisition (lesion in contact or at less than 1 cm of the pleura) and if the investigator estimated that the diagnosis could be reasonably reached with the forceps TBB only (biopsy tool located inside or in front of the lesion on CBCT reconstruction).

Biopsy specimens were fixed in 10% formalin and embedded in paraffin. Hematoxylin and eosin as well as Masson's Trichrome, Giemsa staining were performed as well as immunostaining against pancytokeratins.

Within three hours after the procedure, a chest radiograph was obtained to detect eventual pneumothorax. All patients stayed at hospital for one night after the procedure for monitoring in order to identify relapse of bleeding and subacute pneumothorax.

### Collected data

Demographic information, lung function tests, nodule attenuation and size, sPAP, duration of procedure, TBB and TBLC pathological results, result of any subsequent surgical or non-surgical biopsy, adverse events, length of hospital stay, and post-operative follow-up were collected. The final diagnosis was determined after a minimum of 12 months of follow up. It allowed to determine the true diagnosis (malignant or nonmalignant). All cases were followed according to the practitioner’s judgment (e.g., surgical tissue biopsy, CT-guided transthoracic needle biopsy, serial CT imaging, and lung health visits). Unconclusive ENB cases with subsequent diagnostic tests confirming a nonmalignant diagnosis or without lesion progression on radiographic follow-up were considered true-negative (TN) for malignancy. Alternatively, if follow-up diagnostic test revealed malignancy or growth of the lesion or if patients underwent a stereotaxic radiotherapy (high probability of neoplasia), unconclusive ENB result was considered as false-negative (FN).

Bleeding was assessed using an adapted scoring system previously used by our group for bleeding following TBLC [8] as follow: 0 if absent, 1 (mild) if stopped with aspiration only and/or insufflation of the Fogarty balloon less than five minutes, 2 (moderate) if cold saline was used to control the bleeding and/or the Fogarty balloon needed to be inflated more than five minutes, and 3 (severe) if any of the following treatments was required: embolization, selective bronchial intubation, transfusion, admission in intensive care unit (ICU), or resulting in death or prolonged hospital stay.

### Endpoints

The primary endpoint was the comparison of the diagnostic yield and the diagnostic accuracy obtained in the two arms of the study (endoscopy suite vs CBCT suite). The diagnostic yield was defined as the proportion of patients of each group with a definite pathological diagnosis (malignant or non-malignant) following the ENB procedure compared to the corresponding total number of patients. Of note, patients without diagnosis after the ENB and a minimum follow up of 12 months were not excluded from the determination of the diagnostic yield. The diagnostic accuracy was calculated as the rate of true-positives (for malignancy) (TP) plus true-negatives (for malignancy) (TN) of all subjects with attempted lung lesion biopsies. Patients with a lost to follow up were excluded from the determination of the diagnostic accuracy. However, these cases were further included in a sensitivity analysis, assuming all were FN and then TN, to provide low and high estimates of the diagnostic accuracy.

Secondary endpoints included sensitivity defined as the proportion of positives that are correctly identified as such (TP/TP + FN), specificity defined as the proportion of negatives that are correctly identified as such (TN/TN + FP), positive predictive value defined as TP/(TP + FP), and negative predictive value defined as TN/(TN + FN). We determined also in each group the rate of adequate location of the endoscopic tool within the targeted lesion (“lesion reached”) defined by the combination of the presence of abnormal lung parenchyma (with or without a clear pathological diagnosis), and a confirmation of the adequate localization of the biopsy tool within the nodule on the basis of the result of r-EBUS, CBCT or fluoroscopy acquisition. Other secondary endpoints include safety data, duration of procedures, diagnostic yields of forceps TBB and cryobiopsy (globally and in each arm separately).

### Statistical considerations and analysis

The number of participants in the study was determined using the online application BiostaTGV with an error α of 5% and a power (1-β) of 80%, and an expected difference of diagnostic yield of 30%. According to the D’Agostino & Pearson test used to challenge the normality of the distributions; comparisons between two groups were tested by unpaired Student t tests or Mann–Whitney tests. Proportions were compared using the Chi^2^ test. Statistical analyses were performed using GraphPad Prism 6 (GraphPad Software, La Jolla, CA, USA). For all tests, a *P*-value of less than 0.05 was considered statistically significant.

## Results

**Fig. 1:**
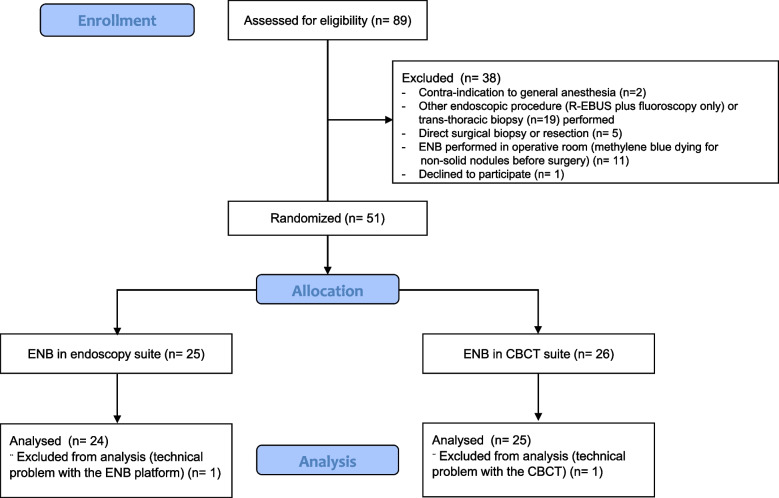
CONSORT flow diagram of the study. CBCT: cone beam computed tomography; ENB: electromagnetic navigation bronchoscopy; R-EBUS: radial endobronchial ultrasound

Between February 2020 and December 2021, 89 patients were screened and 51 patients randomized. Among these, the ENB procedure could be performed as per protocol required in 24 patients in the Endoscopy suite and 25 patients in the CBCT suite, as shown in Fig. 1. A previous unconclusive diagnostic procedure was performed in 19 patients (39%) before their inclusion in the study (TBB: *n* = 11, EBUS: *n* = 3, bronchoalveolar lavage: *n* = 8). Each patient had a single ENB procedure targeting one nodule. The main clinical and nodules characteristics are summarized in Table 1. No significant differences were noticed between the two groups. Nodules were solid in a majority of the cases (75%) and their mean size was 15,9 ± 4,6 mm vs 16,6 ± 6,0 mm for patients in the Endoscopy or CBCT suites respectively (*p* = NS). Final pathological diagnosis based on TBB, cryobiopsy or further surgical biopsies confirmed malignancies in 73% of the cases (specially lung adenocarcinoma) (Table 2). A cryptogenic organizing pneumonia (COP) was identified in two patients. The diagnostic of COP (and the absence of associated malignancy) was further confirmed during the follow up period showing migration of nodules and/or spontaneous decrease in size. 8 patients remained with an undetermined pathological diagnosis after a follow up period of 12 months. Among these, four were considered as probably related to a malignancy and had a stereotaxic radiotherapy.

**Table 1:** Clinical and nodules characteristics of the patients included in the study

Patients and nodules characteristics	All (*n* = 49)	Endoscopy (*n* = 24)	CBCT (*n* = 25)	*P* value
Age mean (SD)	65 (10)	65 (11)	64 (10)	0.864
Gender (woman) %	54	58	48	0.469
BMI mean (SD)	25 (4)	25 (4)	26 (4)	0.332
Smoking (tobacco) %				
Active	18	21	16	0.662
Past	64	58	68	0.483
Never	18	21	16	0.662
PFT mean (SD)				
% FEV	75 (21)	74 (21)	77 (20)	0.676
% FVC	85 (20)	87 (16)	84 (23)	0.664
% DLCO	60 (21)	64 (25)	56 (17)	0.269
sPAP (mmHg) mean (SD)	31 (6)	30 (7)	31 (6)	0.860
Size (mm) mean (SD)^a^	16,3 (5,3)	15,9 (4,6)	16,6 (6,0)	0.659
Type of nodule %				
Solid	76	79	72	0.560
Part-solid	14	12	16	0.726
Non-solid	10	9	12	0.706
Bronchus sign %	39	37	40	0.858
Tangential bronchus %	24	33	16	0.158
Mean distance to pleura (mm)	17.5	17.3	17.7	0.895
Localisation %				
RUL	39	29	48	0.176
ML	6	8	4	0.527
RLL	12	13	12	0.957
LUL	29	29	28	0.928
LLL	14	21	8	0.199

Statistical analysis (chi^2^ or student T-tests) compared the Endoscopy group to the CBCT group

^a^Size is defined as the maximum transverse diameter measured on chest CT scan

*BMI* body mass index, *DLCO* diffusing capacity for carbon monoxide, *FEV* forced expiratory volume, *FVC* forced vital capacity, *PFT* pulmonary function test, *sPAP* systolic pulmonary artery pressure, *RUL* right upper lobe, *ML* middle lobe, *RLL* right lower lobe, *LUL* left upper lobe, *LLL* left lower lobe

**Table 2:** Final pathological diagnosis. COP indicates cryptogenic organizing pneumonia

Final pathological diagnosis	All patients (*n* = 49)	Endoscopy (*n* = 24)	CBCT (*n* = 25)
**Malignant lesion**	36	18	18
Adenocarcinoma	24	12	12
Epidermoid carcinoma	4	2	2
Small cell lung carcinoma	1	0	1
Melanoma (metastasis)	2	2	0
Breast cancer (metastasis)	2	0	2
Colon cancer (metastasis)	1	1	0
Carcinoid tumor	2	1	1
**Benign lesion**	5	2	3
COP	2	1	1
Tuberculosis	2	0	2
Sarcoidosis	1	1	0
**Unspecific or normal lung**	8	4	4

**Fig. 2:**
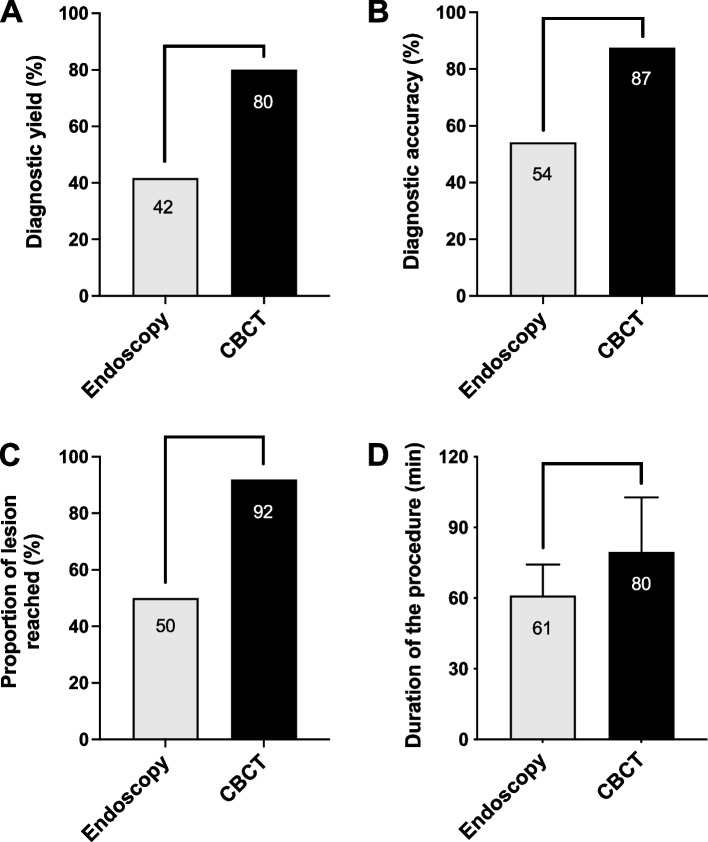
Comparison of diagnostic yield (**A**), diagnostic accuracy for malignancy (**B**), proportion of lesion reached (confident positioning of the catheter based on a radiological confirmation of the catheter within the lesion by r-EBUS, fluoroscopy or CBCT acquisition) and abnormal tissue at pathological examination of biopsy samples) but without definitive pathological diagnosis (**C**), and duration of the procedure (**D**) between patients of both groups. *: *p* < 0,05; **: *p* < 0,01

Regarding the primary endpoint, the diagnostic yield of ENB performed in the CBCT suite was significantly higher than those performed in the endoscopy suite (80% vs 42%, *p* = 0.023) (Fig. 2 and Table 3). The diagnostic accuracy for malignancy was also significantly higher in the CBCT suite than in the endoscopy suite (87% vs 54%, *p* = 0.039) One patient in the CBCT group was excluded from the determination of the diagnostic accuracy according to a lost to follow up and was further included in a sensitivity analysis. Assuming it was a false-negative and then a true-negative case, the comparison of the diagnostic accuracy for malignancy among groups persistently showed significant differences (84% vs 54% and 88% vs 54% respectively, *p* < 0,05). The positive predictive value was 100% in both groups whereas the negative predictive value was 62 and 27% for the CBCT or endoscopy suite respectively (*p* = 0,093) (Table 3). We also determined the proportion of nodules considered as reached even if no specific pathological was obtained (confident positioning of the catheter based on r-EBUS, fluoroscopy or CBCT acquisition and abnormal tissue at pathological examination of biopsy samples). This proportion was 92% vs 50% for procedures performed in CBCT or Endoscopy suites respectively (*p* = 0.001) (Fig. 2 and Table 3).

**Table 3:** Primary and secondary endpoints

		**All procedures**	**Endoscopy**	**CBCT**	*P* value *
**Primary endpoint**	**Diagnostic yield**	61%	42%	80%	**0.023**
		(30/49)	(10/24)	(20/25)	
**Secondary endpoints**	**Diagnostic accuracy**	71%	54%	87%	**0.039**
		(34/48)	(13/24)	(21/24)	
	**Sensitivity**	64%	45%	84%	**0.011**
		(25/39)	(9/20)	(16/19)	
	**Specificity**	100%	100%	100%	> 0,999
		(9/9)	(4/4)	(5/5)	
	**PPV**	100%	100%	100%	> 0,999
		(25/25)	(9/9)	(16/16)	
	**NPV**	39%	27%	62%	0.093
		(9/23)	(4/15)	(5/8)	
	**Lesion reached**	71%	50%	92%	**0.001**
		(35/49)	(12/24)	(23/25)	
	**Additional information from TBLC**	14%	12.5%	17%	0.703
	(undiagnostic TBB but diagnostic TBLC)	(6/42)	(3/24)	(3/18)	
	**Additional information from TBB**	17%	12.5%	22%	0.403
	(undiagnostic TBLC but diagnostic TBB)	(7/42)	(3/24)	(4/18)	
	**Duration of procedures (minutes)**	70 (21)	61 (13)	80 (23)	**0.001**
	Mean (SD)				
	**Pneumothorax**	6%	8%	4%	0.819
		(3/49)	(2/24)	(1/25)	
	**Bleeding**				
	No or mild (grade 1)	90%	83%	96%	0.845
		(44/49)	(20/24)	(24/25)	
	Moderate (grade 2)	10%	17%	4%	0.143
		(5/49)	(4/24)	(1/25)	
	Severe (grade 3)	0%	0%	0%	> 0,999
		(0/49)	(0/24)	(0/25)	

Data are represented as % (n/total) except for the duration of the procedure (mean (SD))

*NPV* negative predictive value, *PPV* positive predictive value, *TBB* trans-bronchial biopsy, *TBLC* trans-bronchial lung cryobiopsy. Statistical analysis (chi^2^ or student T-tests) compared the Endoscopy group to the CBCT group

The duration of the procedure was significantly higher in the CBCT group than in the Endoscopy group (80 ± 23 min vs 61 ± 13 min respectively, mean ± SD, *p* < 0,01) (Fig. 2 and Table 3). In the CBCT group, the median number of scans was 3 (ranging from 1 to 8) with a cumulative ionizing radiation exposure of 5,6 ± 2,6 (1,7–10,9) mSv, mean ± SD (range). The ENB catheter was repositioned in 20 of the 25 CBCT cases. In two patients, it was not possible to access the lesion with the ENB catheter only and a CrossCountry procedure was performed. It allowed to reach the lesion and obtain a diagnosis in one case. In those two patients, the duration of the procedure was 130 and 75 min and respectively 8 and 3 CBCT acquisitions were performed.

Trans-bronchial lung cryobiopsies (TBLC) were performed in 42 patients (all patients in the endoscopy group and 18/25 (72%) of the patients in the CBCT group). Indeed, we decided not to perform TBLC in some patients of the CBCT group if nodule appeared closer than expected to the pleura during CBCT acquisition and if we estimated that diagnosis could be reasonably reached with the forceps TBB only. The corresponding diagnostic yield in those patients was 86%. Also, the benefit to perform both type of biopsies was calculated. The added value to perform TBLC on top of TBB with forceps (*i.e.* the number of TBLC that are positive when the TBB is negative) was 14% without difference among groups (12,5% and 17% respectively; *p* = NS). Similarly, the added value to perform TBB in addition to TBLC was 17%, again without difference regarding groups (12,5% vs 22% respectively; *p* = NS) (Table 3).

Regarding adverse events, pneumothorax occurred in 3 patients out of 49 (6%) without difference regarding groups (2/24 (8%) vs 1/25 (4%) for procedures performed in the endoscopy suite or under CBCT guidance respectively; *p* = NS) (Table 3). Two pneumothoraxes were not detected in the chest X-ray performed in the recovery room but later during the overnight monitoring period. The two patients having a CrossCountry procedure did not develop a pneumothorax. No severe bleeding was observed. When bleeding occurred, it was mild in a majority of the cases (65%), again without difference among groups (16/24 (67%) vs 16/25 (64%) respectively; *p* = NS). Interestingly, no relapse of bleeding occurred during the overnight monitoring period.

Finally, the impact on the diagnostic yield of characteristics of the nodule was analyzed. This included nodule size, real-time visualization of the nodule with the fluoroscopy, presence of a bronchus sign and the result of the rEBUS evaluation performed in all patients except four for technical problems (one in the endoscopy group and three in the CBCT group). No significant differences were observed for any of these parameters considering the overall population or each study group (endoscopy vs CBCT) (Table 4).

**Table 4:** Diagnostic yields according to nodules characteristics and endoscopic procedure

	**Diagnostic yield**											
	Overall population (*n* = 49)											
	**Nodule size**			**Bronchus sign**			**Visibility at fluoroscopy**			**R-EBUS positivity**		
	< 20 mm	20–30 mm	*P* value	Yes	No	*P* value	Yes	No	*P* value	Yes	No	*P* value
**All (%)**	23/36 (64)	7/13 (54)	0.524	12/19 (63)	18/30 (60)	0.825	8/14 (57)	22/35 (63)	0.711	24/37 (65)	4/8 (50)	0.432
**Endoscopy (%)**	8/18 (44)	2/6 (33)	0.633	5/9 (55)	5/15 (33)	0.285	5/10 (50)	5/14 (36)	0.484	8/16 (50)	1/5 (20)	0.237
**CBCT (%)**	15/18 (83)	5/7 (71)	0.504	7/10 (70)	13/15 (87)	0.307	3/4 (75)	17/21 (81)	0.785	16/21 (76)	3/3 (100)	0.342

## Discussion

This prospective randomized monocentric trial aimed to investigate the added value of performing ENB procedure under CBCT guidance rather than in a standard endoscopy suite. Our results confirmed this benefit as the diagnostic yield almost double when ENB was performed under CBCT guidance (80% instead of 42%). Also, the diagnostic accuracy was higher under CBCT guidance (87% instead of 54%), a difference remaining significant in the sensitivity analysis. This high diagnostic yield and accuracy is in line with previously published data (ranging from 75 to 83%) for ENB procedures performed under CBCT guidance for small-sized nodule (less than 2 cm) [4, 12]. This advantage is probably explained by the real-time verification of the localization and axis of the catheter and biopsy tools during CBCT scans, repositioning of the catheter if necessary and by the use of enhanced fluoroscopy permitting to highlight the lesion in addition to standard fluoroscopy images.

The high diagnostic yield observed under CBCT guidance in our study was obtained by the combined use of both forceps TBB and TBLC, each of them increasing the diagnostic yield equally (± 15%). Of note, the diagnostic yield of 42% obtained in the endoscopy suite under fluoroscopic guidance only appeared lower than previously reported [4–8]. However, this apparent inferiority is probably explained by multiple factors. Firstly, the definition used for the diagnostic yield differs among studies. For example, in the NAVIGATE study, the diagnostic yield reported correspond rather to the definition of the diagnostic accuracy used in our study. Therefore, for lesion of mean size of 20 mm (higher than in our study), the diagnostic yield was 72% which must be compared to our diagnostic accuracy of 54% [7]. Interestingly, in lesion of similar size than in our study, Verhoeven and colleagues reported a diagnostic accuracy of 50% in their subgroup with ENB alone, in line with our data [4]. Also, by applying the same definition than in our study, the diagnostic yield of the NAVIGATE study became 50% which seems relatively similar to our result taking into account that we obtained this on smaller nodule (49% of the nodules in the NAVIGATE study versus 73% of the nodule in our study were smaller than 2 cm). Secondly, we performed a very high selection of candidate patients. Indeed, in Belgium, ENB is not reimbursed and could only be performed thanks to a financial support from a scientific foundation of our institution. Therefore, according to this limited budget and a growing demand for undiagnosed nodules, included patients were rigorously selected. Specially, if another endoscopic or trans-thoracic biopsy could provide the diagnosis, this has to be used first. Also, nodules with a high probability of neoplasia in operable patients (suspected Stage 1 and 2) were preferentially referred to surgery directly [11, 13, 14]. Consequently, only 51 of the 89 patients screened were included and 40% of them already had a prior diagnostic bronchoscopy. Altogether, these factors explain the relatively low diagnostic yield observed for ENB performed in the endoscopy suite.

CBCT scans also provided interesting information regarding the distance of the nodule from the chest wall that could differ from pre-procedural chest CT scan due to lower level of pulmonary inflation and the development of atelectasis under general anesthesia. Accordingly, if peripheral lesion appeared closer than expected to the pleura during CBCT acquisition, TBLC were not performed if the diagnosis could be reached with TBB only (forceps located inside or in front of the lesion on CBCT acquisition). Theoretically, this could possibly reduce the risk of pneumothorax following TBLC when performed under CBCT guidance than under standard X-ray fluoroscopy. However, according to the low number of pneumothorax in our study (*n* = 3) no conclusion regarding possible reduction in the rate of pneumothorax when procedures were performed under CBCT guidance could be made. Finally, another potential advantage of CBCT guidance is to allow the use of trans-parenchymal access tools such as the CrossCountry catheter [9, 10]. This technique was also used in our study but, again, in a too limited number of patients (*n* = 2) to drive conclusions on its added value.

The added value of CBCT guidance in addition to ENB to reach targeted lesions was somewhat counterbalanced by a mild increase in the duration of the procedure as observed in our study. In addition, the accessibility to such hybrid room and the cost related to the combined used of CBCT and ENB could be another limitation in some centers. Also, higher ionizing radiation exposure compared to X-ray fluoroscopy could represent another disadvantage of CBCT guidance. Unfortunately, our study cannot evaluate this latter point as we did not systematically record this exposure for ENB performed in the endoscopy suite under standard X-ray fluoroscopy guidance. However, the radiation exposure observed in the CBCT group (5,6 mSv/patient), which is in line to the exposure reported in a similar study using ENB under CBCT guidance [15], remained higher than the exposure reported for biopsy performed under X-ray fluoroscopy (0,49 mSv) [16].

Our study presents several limitations. It is a single-center (monocentric) and unblinded study. The number of subjects did not allow to perform accurate subgroups analysis such as correlations between nodules characteristics (size, presence of a bronchus sign, visibility on standard fluoroscopy) and the diagnostic yield or to demonstrate an added value to use the CrossCountry technique. For example, in the CBCT group, three patients had a surprising negative rEBUS whereas all of them had pathological diagnosis following biopsies. At the opposite, the diagnostic yield was only 76% when rEBUS was positive. However, no conclusion on the benefit to perform or not rEBUS when ENB is performed under CBCT guidance could be made according to the low number of patients in this subgroup analysis. Similarly, nodule size was previously shown as a factor influencing the diagnostic yield [5, 7]. However, in our study, no effect of the size was noticed. There was even a trend for higher diagnostic yield when the diameter was lower than 2 cm. Again, this is probably explained by the low number of patients and a low variability in the size of already small nodules (16 mm).

Also, Medtronic developed in the meantime a new ENB platform called illumisite™. This platform uses tomosynthesis to correct the virtual target reducing the discrepancy between the static CT scan and the dynamic breathing lung, enhancing the visibility of the nodule on X-ray fluoroscopy, and allowing to locally register the nodule and adjust alignment during the procedure [17, 18]. Accordingly, Aboudara and colleague showed in a retrospective study on 168 cases a diagnostic yield of 79% using the illumisite™ platform compared to 54% with the SuperDimension one [19]. It is therefore possible that the advantage of the CBCT guidance could be less pronounced using this new ENB platform.

In conclusion, we showed that the use of CBCT guidance during ENB procedure for nodule of a mean size less than 2 cm significantly increases the diagnostic yield of the technique. Even more than 90% of the lesion could be reached by the biopsy tools, opening new perspectives for future therapeutic approach of these nodules as bronchoscopy-guided ablation.

## Data Availability

The datasets used and/or analyzed during the current study as well as the full protocol are available from the corresponding author on reasonable request.

## Abbreviations

BMI: Body mass index
CBCT: Cone beam computed tomography
COP: Cryptogenic organizing pneumonia
DLCO: Diffusing capacity for carbon monoxide
ENB: Electromagnetic navigation bronchoscopy
FEV_1_: Forced expiratory volume in 1 s
FVC: Forced vital capacity
GGO: Ground glass opacity
HRCT: High-resolution computed tomography
ICU: Intensive care unit
LDCT: Low dose computed tomography
PEEP: Positive end-expiratory pressure
PFT: Pulmonary function test
rEBUS: Radial endobronchial ultrasound
sPAP: Systolic pulmonary artery pressure
TBB: Trans-bronchial biopsy
TBLC: Transbronchial lung cryobiopsy
